# Detection of La Crosse Virus In Situ and in Individual Progeny to Assess the Vertical Transmission Potential in *Aedes albopictus* and *Aedes aegypti*

**DOI:** 10.3390/insects14070601

**Published:** 2023-07-03

**Authors:** Christie S. Darby, Kyah M. Featherston, Jingyi Lin, Alexander W. E. Franz

**Affiliations:** Department of Veterinary Pathobiology, University of Missouri, Columbia, MO 65211, USA; christieherd87@gmail.com (C.S.D.); kmfeatherston@mail.missouri.edu (K.M.F.); lynn.sitrun@gmail.com (J.L.)

**Keywords:** La Crosse virus, *Aedes albopictus*, *Aedes aegypti*, vertical transmission, vector competence, ovary, follicle, nurse cell, bloodmeal, progeny, oviposition

## Abstract

**Simple Summary:**

We investigated the vertical transmission potential of the *Orthobunyavirus* La Crosse virus (LACV) in the mosquitoes *Ae. albopictus* and *Ae. aegypti.* In both mosquito species, productive midgut infection with the virus was a prerequisite for the following ovary infection. However, the midgut infection levels varied between both species, with *Ae. albopictus* being more susceptible to LACV infection than *Ae. aegypti*. In both mosquito species, the vertical transmission rates of LACV from mothers to offspring were below those levels typically described for transovarial transmission (TOT). The LACV infection patterns in the ovarian tissue of *Ae. aegypti* suggested the transovum transmission of the virus. In several *Ae. albopictus* samples, LACV antigen was detected in follicular tissue or in a few developing oocytes, indicating that the TOT of LACV could be potentially occurring in this mosquito species. Thus, TOT is not a general feature of LACV infections in mosquitoes.

**Abstract:**

La Crosse virus (LACV) is circulating in the midwestern and southeastern states of the United States and can cause human encephalitis. The main vector of the virus is the eastern tree-hole mosquito, *Aedes triseriatus*. *Ae. albopictus* has been also described as a natural LACV vector, while *Ae. aegypti* has been infected with the virus under laboratory conditions. Here, we compare the vertical transmission potential of LACV in *Ae. albopictus* and *Ae. aegypti*, with emphasis given to the ovarian infection patterns that the virus generates in both species. Both mosquito species received artificial bloodmeals containing LACV. At defined time points post-infection/bloodmeal, midguts, head tissue, and ovaries were analyzed for the presence of virus. Viral infection patterns in the ovaries were visualized via immunofluorescence confocal microscopy and immunohistopathology assays using an LACV-specific monoclonal antibody. In *Ae. aegypti*, LACV was confronted with midgut infection and escape barriers, which were much less pronounced in *Ae. albopictus*, resulting in a significantly higher prevalence of infection in the latter. Following the ingestion of a single virus-containing bloodmeal, no progeny larvae were found to be virus-infected. Regardless, females of both species showed the presence of LACV antigen in their ovariole sheaths. Furthermore, in a single *Ae. albopictus* female, viral antigen was associated with the nurse cells inside the primary follicles. Following the ingestion of a second non-infectious bloodmeal at 7- or 10-days post-ingestion of an LACV-containing bloodmeal, more progeny larvae of *Ae. albopictus* than of *Ae. aegypti* were virus-infected. LACV antigen was detected in the egg chambers and ovariole sheaths of both mosquito species. Traces of viral antigen were also detected in a few oocytes from *Ae. albopictus*. The low level of vertical transmission and the majority of the ovarian infection patterns suggested the transovum rather than transovarial transmission (TOT) of the virus in both vector species. However, based on the detection of LACV antigen in follicular tissue and oocytes, there was the potential for TOT among several *Ae. albopictus* females. Thus, TOT is not a general feature of LACV infection in mosquitoes. Instead, the TOT of LACV seems to be dependent on its particular interaction with the reproductive tissues of a female.

## 1. Introduction

La Crosse virus (LACV) (family: *Peribunyaviridae*; genus: *Orthobunyavirus*) belongs to the California serogroup viruses and is a leading cause of pediatric encephalitis in the United States [1]. LACV was initially isolated in 1964 from the brain and spinal tissue of an infant who was diagnosed with “rural encephalitis” in 1960 [2]. General symptoms, such as fever, lethargy, and vomiting, can develop 5–15 days after infection with the virus, but a severe neuroinvasive disease outcome, particularly in people under the age of 18 years is possible [3]. From 2003 to 2019, 92% of 1281 reported human LACV cases were neuroinvasive. Cases of LACV commonly occur in states spanning from the upper Midwest to the southeast United States, with 80% of the cases historically occurring in West Virginia, North Carolina, Ohio, and Tennessee [4]. Chipmunks and squirrels in oak hickory forests represent the principal animal reservoir for LACV in horizontal transmission cycles during summer and fall, while during winter, the virus survives in diapausing mosquito eggs [5,6]. The eastern tree-hole mosquito, *Aedes triseriatus*, is the primary vector of LACV, and it is typically found in forested areas in the eastern and central regions of the United States [7,8,9]. LACV has also been detected in mosquito pools of other *Aedes* species. For example, LACV-positive *Ae. cinereus*, *Ae. canadensis*, and *Ae. trivittatus* were found in Connecticut in 2018 [10]. Furthermore, field samples of *Ae. albopictus* have been found to be LACV-infected [11,12]. *Ae. triseriatus* transmits LACV horizontally via the oral and venereal routes and vertically through the transovarial route [13,14,15,16]. Similar to other arboviruses, LACV first needs to infect the mosquito midgut before it can disseminate to secondary tissues, including the fat body, neural tissue, ovaries, and salivary glands. Productive salivary gland infection is critical for effective horizontal virus transmission to the vertebrate host, whereas vertical transmission depends on the infection of the reproductive tissues in the female mosquito. 

The reproductive organ of a female *Ae. aegypti* mosquito consists of two spindle-shaped ovaries with two lateral oviducts joining the median oviduct [17]. Each ovary contains approximately 100 polytrophic meroistic ovarioles in which stem cells, primary oocytes, prefollicular tissues, and nurse cells are contained. The clustered ovarioles are held together in each ovary by a mesh of muscular sheaths, including the tunica externa [17,18]. The ovarioles are attached to the muscular ovarian sheath by their germarial end and enveloped by the ovariole sheath, which becomes progressively thinner during egg development. The ovariole sheath consists of a cellular epithelial sheath on the outside and an amorphous tunica propria on the inside. By being associated with the follicle on one side and the fat body on the other side, the ovariole sheath acts as a filter for incoming hemolymph proteins from the fat body to the follicles [19,20]. Each ovariole contains isolated oogonia in the terminal end chamber and two developing follicles (egg chambers) towards the oviduct in the following order: oogonium–secondary follicle–primary follicle [17,21]. Each ovariole sheath is continuous with the outer layer of the calyx, while the lateral oviduct is surrounded by a circular muscle sheath, which is continuous with the ovarian sheath [22]. The structures of both the lateral oviduct and calyx are modified after initial bloodmeal ingestion by the mosquito. The ovarian follicles develop synchronously throughout oogenesis. The secondary follicles contain undifferentiated nurse cells. In the primary follicle, between 12 and 24 h post-bloodmeal (pbm), the oocyte can be distinguished from the nurse cells, which transport newly synthesized mRNA, structural proteins, and eventually their own organelles (ribosomes, mitochondria) to the oocyte during its pre-vitellogenic growth [23]. Once most of their cytoplasmic content has been translocated into the developing oocytes, the nurse cells degenerate [24]. In the primary follicle, each oocyte in the vitellogenic stage is surrounded by a layer of follicular epithelial cells, which are mainly responsible for secreting the macromolecules used for chorion formation. Vitellogenin comprises yolk protein precursors, which are produced outside the ovaries. The follicular uptake of vitellogenin is regulated by steroid hormones and requires the presence of vitellogenin receptors. At 40 h pbm, the chorion is deposited by the follicular epithelium, while at 48 h pbm, the follicular epithelial cells start to disintegrate. Oviposition then starts at 56–60 h pbm and may last up to 88 h pbm [23]. After oviposition, remnants of the follicular epithelium, containing many lysosomes, remain attached to the calyx [22].

Vertical arbovirus transmission by female mosquitoes can occur by two distinct pathways: either through the transovarial or transovum route [1]. Transovarial virus transmission (TOT) involves the infection of the ovarian follicles, causing the mosquito progeny to be infected as well. In contrast, during transovum transmission, the progeny becomes infected via the micropyle during oviposition when the oocyte is in close contact with the virus-infected ovarian calyx and oviduct [25,26,27]. TOT is considered to be more efficient in terms of the number of progeny (originating from a single female) to be infected compared to transovum transmission. TOT by mosquitoes of the genera *Aedes* and *Culex* has been described for the negative-sense RNA virus Rift Valley fever virus (RVFV) (family: *Phenuiviridae*, genus: *Phlebovirus*) and several viruses belonging to the *Peribunyaviridae* (genus: *Orthobunyavirus*), including San Angelo virus (SAV), snowshoe hare virus, Tahyna virus [28], and LACV [1,29]. Furthermore, TOT by *Aedes* spp. has been reported in recent years for flaviviruses (family: *Flaviviridae*; genus: *Flavivirus*), such as dengue virus (DENV) and Zika virus [30,31,32,33,34,35,36]. Vertical transmission has been also reported for the alphaviruses (family: *Togaviridae*; genus: *Alphavirus*) Sindbis virus and chikungunya virus [32,37]. 

LACV has become a model system for the study of TOT by its principal vector, *Ae. triseriatus* [1]. However, so far, only a single in situ detection study has been conducted for LACV showing ovarian infection of the virus in its vector indicative of TOT [38]. In this study, we examined the vertical transmission potential of LACV by *Ae. aegypti* and *Ae. albopictus* using artificial virus challenge assays in combination with quantitative and visual detection techniques based on immunofluorescence and immunohistochemistry assays. Our data show a difference in the overall vector competence for LACV between *Ae. aegypti* and *Ae. albopictus*. Moreover, in both mosquito species, the predominantly observed vertical transmission pattern of the virus was not in accordance with TOT. 

## 2. Materials and Methods

### 2.1. Mosquito Maintenance 

Two laboratory-adapted *Aedes* species were used for the experiments involving horizontal LACV transmission: *Aedes albopictus* Lake Charles (LC) [39] and *Ae. aegypti* Higgs White Eye (HWE) [40]. Larvae of both species were reared in shoe-box-size containers filled with 800 mL of distilled water and provided with tropical fish food (Tetra, Melle, Germany) ad libitum until pupation. Pupae were placed in water cups inside cardboard containers, which were covered with a mesh netting. Following eclosion, the pupa cups were removed while raisins and small water cups were placed onto the netting of the cartons as nutrient source for the adults. Mosquitoes were maintained in a humidified insectary at 28 °C and 80% humidity under a 12 h light/dark cycle.

### 2.2. Preparation of LACV-Containing Artificial Bloodmeals 

We used LACV (VR-1834, ATCC) for our mosquito challenge experiments, which was originally isolated in 1964 [2]. The virus was propagated in BHK-21 cells (CCL-10, ATCC), which were grown in Dulbecco’s Modified Eagle Medium (DMEM) supplemented with 7% fetal bovine serum (FBS). At 90% confluency, cells were infected with LACV at an MOI of 0.01. Cell supernatant was collected at 48 h post-infection, or when 60% of the cells showed CPE. Virus-containing culture supernatant was mixed with defibrinated sheep blood (Colorado Serum Company, Denver, CO, USA) at a 1:1 ratio and provided to the 5–6-day-old females inside a carton through a glass feeder (1 feeder per carton) covered with a parafilm membrane. The glass feeders were heated by a water jacket to 37 °C. Mosquitoes were allowed to feed on the virus-containing meal for 45 min, after which they were anaesthetized on ice to select for engorged females. These were maintained in a humidified chamber at 28 °C and 80% humidity in cardboard cartons and supplied with raisins and water cups until sampled. LACV bloodmeal titers ranged from 8.0 × 10^5^ to 1.3 × 10^6^ plaque-forming units per ml (PFUs/mL). 

### 2.3. Plaque Assays for the Detection of LACV

At defined time points following ingestion of an LACV-containing bloodmeal, midguts, head tissue, and ovaries were dissected from mosquitoes and separately homogenized in 0.5 mL of DMEM (supplemented with 7% FBS and 5% HEPES) using a hand-held homogenizer. Samples were then filtrated using 0.22 μM Supor Membrane syringe filters (Pall Life Sciences, East Hills, NY, USA). Filtered samples were 10-fold diluted in 96-well plates, and 150 μL of each sample from each well of the dilution series was transferred to confluent Vero cells in 24-well-plate format. Cells were incubated for 1 h at 37 °C and under 5% CO_2_ supplement, while rocking every 15 min. Cells were then overlaid with a 1% agarose and nutrient mixture consisting of 10% M199 (10×), 7% FBS, 0.5% MEM non-essential amino acids (100×), 0.5% MEM vitamin solution (100×), and 0.003% sodium bicarbonate (Gibco, ThermoFisher Scientific, Waltham, MA, USA). Plates were incubated at 37 °C under 5% CO_2_ supplement for 3 days, before staining with 500 mL/well MTT (3-[4,5-dimethylthiazol-2-yl]-2,5-diphnyltetrazolium bromide) (Sigma-Aldrich, St. Louis, MO, USA), followed by incubation at 37 °C for 24 h. Plaque numbers were counted for each sample, and viral titers of individual tissues were calculated as PFUs/mL.

### 2.4. Immunofluorescence Confocal Microscopy (IFCM) to Detect LACV Antigen in Mosquito Tissues 

Midguts and ovaries were dissected at various time points following challenge with an LACV-containing bloodmeal and fixed in 4% paraformaldehyde–phosphate buffer solution (PBS) (Gibco, ThermoFisher Scientific) at 4 °C for a minimum of 30 min. Tissues were permeabilized in PBS-T (PBS, 1% BSA, 0.2% Triton X-100) at room temperature (RT) for 1 h. Tissues were then incubated overnight at 4 °C in PBS-T containing LACV monoclonal antibody (8C2.2) (Invitrogen, ThermoFisher Scientific (Waltham, MA, USA)) at a 1:200 dilution. Samples were then washed three times for twenty minutes each with PBS-T, then incubated with fluorescent-labeled secondary antibody (Alexa Fluor 594, Abcam: ab150120, Cambridge, UK) at a dilution of 1:500 and counter-stained with Phalloidin (AlexaFluor 488, Invitrogen, ThermoFisher Scientific) at 1:100 dilution for 1 h at RT. Cell nuclei were stained with DAPI (Invitrogen) at 1 μg/mL for ten minutes at RT. Tissues were washed three times again with PBS and mounted on six-well slides using Fluoromount G mounting medium (Electron Microscopy Sciences, Hatfield, PA, USA). Tissues were imaged using an inverted spectral confocal microscope (TCP SP8 MP, Leica Microsystems, Wetzlar, Germany) at the Molecular Cytology Core of the University of Missouri. 

### 2.5. Immunohistochemistry (IHC) Assay to Detect LACV Antigen In Situ

*Ae. aegypti* HWE and *Ae. albopictus* LC females received an LACV-containing bloodmeal (BM-1) (titer: 1.3 × 10^6^ PFUs/mL) followed by a non-infectious bloodmeal (BM-2) at 10-days post-BM-1. Mosquito bodies (without legs and wings) were collected at 24 h post-BM-2 and fixed in 10% neutral-buffered formalin at 4 °C for a minimum of 24 h. Mosquito bodies were then embedded in paraffin and sectioned onto microscope slides for IHC staining. Slides were incubated with LACV monoclonal antibody (8C2.2) (Invitrogen) at a dilution of 1:200 and incubated with a Mouse Envision secondary antibody for 20 min. All IHC staining was performed by the MU Veterinary Medicine Diagnostic Laboratory. Slides were imaged using a Leica ICC50 W microscope equipped with a Wi-Fi-enabled camera. 

### 2.6. Oviposition of Individual LACV-Infected Females and Rearing of F1 Larvae for LACV Detection

Female mosquitoes were fed LACV-containing BM-1 at 5-days post-eclosion and were then maintained in bulk at 28 °C and 80% humidity. An egg cup was provided to collect eggs from BM-1. Those eggs were not analyzed any further. At 7- or 10-days post-BM-1, females received BM-2, followed by anaesthetization on ice to select engorged females for oviposition. Individual females were placed in separate cardboard containers with an egg cup and egg paper for oviposition. Each cup was monitored daily, and eggs were collected and dried for 5 days once spotted. At the same time, midguts and ovaries were dissected from any female that had laid eggs and subjected to IFCM for LACV antigen detection. Eggs were counted on the egg paper once dried, then hatched under vacuum and reared to L4 larvae using tropical fish food in a humidified growth chamber at 28 °C and 80% humidity. If LACV was detected in the corresponding female’s ovaries, then each individual larva from that particular female was analyzed for the presence of LACV by TaqMan qRT-PCR. If LACV was not detected in the ovaries using IFCM, then larvae were pooled in groups of 20 to test for LACV infection. 

### 2.7. RNA Extraction and cDNA Synthesis from Larvae 

L4 larvae were collected individually or in pools for homogenization in 250 μL Tri-Reagent (Sigma Aldrich, St. Louis, MO, USA) using a hand-held homogenizer. For pools of 10–20 larvae, 500 μL of Tri-Reagent was used. Samples were left for 10 min in Tri-Reagent at RT to lyse tissues, then 200 μL of chloroform was added and samples were vigorously mixed, then left for 5 min at RT. Samples were centrifuged at 12,000× *g* for 15 min at 4 °C. The resulting supernatant was added to 250 μL isopropanol, with 1μL glycogen was added to help pellet RNA from individual larva samples. Samples were again centrifuged at 12,000× *g* for 10 min at 4 °C to form a pellet, then washed twice with 75% ethanol. Extracted RNA was resuspended in 20–50 μL of distilled water for quantification using a Nanodrop spectrophotometer. Total RNA (600 ng) was used for cDNA synthesis using Protoscript II First Strand cDNA Synthesis Kit (New England Biolabs, Ipswich, MA, USA), following the manufacturer’s instructions. 

### 2.8. LACV TaqMan qRT-PCR 

For the TaqMan qRT-PCR assays, a standard curve based on serial dilutions of a plasmid vector containing an LACV-derived cDNA insert at a known quantity was generated. To produce the LACV-derived cDNA insert, viral RNA was isolated from supernatant of infected BHK-21 cells in which LACV had grown for 48 h. Viral RNA isolation was performed using Tri-Reagent LS (Sigma Aldrich, St. Louis, MO, USA) using standard extraction protocol. cDNA synthesis and PCR were then performed using the SuperScript III OneStep RT-PCR System with Platinum Taq DNA Polymerase (ThermoFisher Scientific, Waltham, MA, USA) and LACV primers (LACV F: AATTGGAGAGTGGCAGGTG; LACV R: CCCACTGTCCCATCCTACAC). The resulting 223 bp LACV S-segment-derived PCR product was cloned into the pCR4 plasmid vector of the TOPO TA Cloning Kit (Invitrogen, Waltham, MA, USA), according to the manufacturer’s protocol.

TaqMan qRT-PCR was performed on cDNA samples of mosquito larvae to detect LACV. A custom-made LACV TaqMan probe (/56-FAM/CAACGATCT/ZEN/TACCATCCACAGA/3IABkfq/) was used at a final concentration of 250 nM in conjunction with LACV-AT-rich primers; (LACV-AT-F: AATAAATCATAATCCTGGAAACAGGAACAACC; LACV-AT-R: AATAAATCATAACAAGGACCCATCGGCTAAA) at final concentrations of 900 nM. cDNA (2 μL) was added to a master mix containing iTaq Universal Probes Supermix (Bio-Rad, Hercules, CA, USA) using the manufacturer’s standard protocol. TaqMan qRT-PCR was performed on a StepOnePlus real-time PCR system (Applied Biosystems, Waltham, MA, USA) using the standard cycling protocol choosing a 60 °C annealing temperature. 

### 2.9. Statistical Analysis 

Statistical analysis was performed in GraphPad Prism vs. 9 and RStudio. Tissue infection data based on viral prevalence were analyzed using Fisher’s Exact Test. Intensity of LACV infection in the two different mosquito species was analyzed using the non-parametric Kruskal–Wallis test, followed by Dunn’s Multiple Comparison test. Fecundity and vertical transmission data were first tested for normal distribution using a Shapiro–Wilk test. If data were normal-distributed, then a T-test was performed for comparison. If data were not normal-distributed, then a Mann–Whitney U-test was performed (Wilcox test). 

## 3. Results

### 3.1. Detection of LACV in Midguts, Head Tissue, and Ovaries of Ae. aegypti and Ae. albopictus, Which Had Acquired a Single Virus-Containing Bloodmeal 

We tested the vector competence of two different mosquito species, the *Ae. aegypti* strain Higg’s White Eye (from now on named “HWE”) and the *Ae. albopictus* strain Lake-Charles (from now on named “LC”) for LACV when acquired via an artificial bloodmeal. 

**Figure 1:**
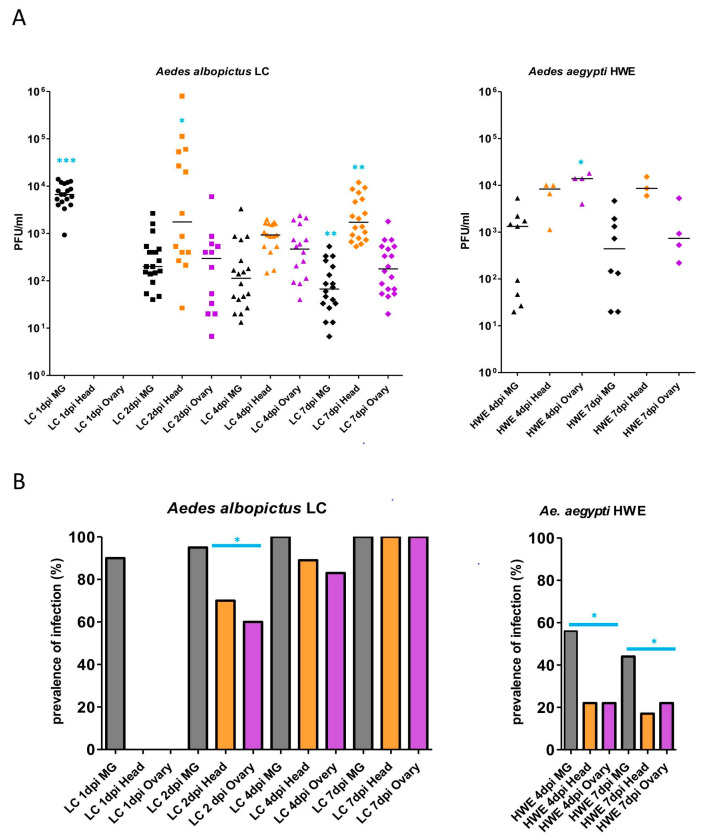
Intensity and prevalence of LACV infection in midgut, head, and ovarian tissues of *Ae. albopictus* LC and *Ae. aegypti* HWE. (**A**) Intensity of LACV infection. Mosquitoes received an artificial bloodmeal containing 8.0 × 10^5^ PFUs/mL LACV (strain from 1964). Midgut, head, and ovarian tissues of each fed female (*n* = from 18 to 20) were analyzed at 1, 2, 4, and 7 dpi (*Ae. albopictus*) and at 4 and 7 dpi (*Ae. aegypti*) for the presence of virus by plaque assays in Vero cells. Intensity of infection was analyzed by the non-parametric Kruskal–Wallis test, followed by Dunn’s Multiple Comparison test. * *p* =/< 0.05; ** *p* =/< 0.01; *** *p* =/< 0.001. (**B**) Prevalence of LACV infection. Mosquitoes received the same bloodmeal as in (**A**). Prevalence of infection was assessed for *Ae. albopictus* LC (*n* = from 19 to 20) and *Ae. aegypti* HWE (*n* = from 18 to 20) using Fisher’s Exact test. * *p* =/< 0.05.

Plaque assay data showed that following the ingestion of an LACV-containing bloodmeal, the virus titer decreased over time in the midguts of LC females, ranging from a median value of ~10,000 PFUs/mL at 1 dpi to <100 PFUs/mL at 7 dpi (*p* = /<0.001) (Figure 1A). The significantly higher titer (*p* = /<0.001) on day 1 may partially reflect the virus in the ingested bloodmeal (8.0 × 10^5^ PFUs/mL), which was unable to infect the midgut before peritrophic matrix formation and eventually was digested along with the bloodmeal. From 2 dpi onwards, LACV was detectable in secondary tissue, such as head tissue (as a proxy for salivary gland infection) and ovaries. Median head tissue titers were relatively steady during the time course at around ~1000 PFUs/mL, albeit significantly increased at 2 dpi (*p* = /<0.05) and 7 dpi (*p* = /<0.01) in comparison to other tissues or time points. The ovarian titers showed more fluctuation, with median titers between >100 and <1000 PFUs/mL, which may reflect the dynamics of ovarian tissue/oocyte development in the female. Median LACV titers in HWE females at 4 and 7 dpi were approximately one magnitude higher than those observed at similar time points for LC (Figure 1A), and significantly higher in the ovaries at 4 dpi (*p* = /<0.05), allowing for the conclusion that, in *Ae. albopictus*, the virus was more strongly controlled and affected by the mosquito’s immune responses than in *Ae. aegypti*. 

At 1 dpi, LACV was detected in 90% of the analyzed LC midguts, but not so in secondary tissues (Figure 1B). One day later, the virus was detected in at least 60% of all three tissues, and at 7 dpi, all three tissues from all assayed LC mosquitoes were LACV-infected. By contrast, in HWE females, the LACV infection rates were only between 15% (head tissue at 7 dpi) and 58% (midgut at 4 dpi), which were significantly less than those observed for LC females (*p* = from /<0.01 to <0.0001) (Figure 1B). Thus, during the seven-day time course, HWE mosquitoes exhibited strong midgut infection and midgut escape barriers for LACV, which were much less pronounced in LC mosquitoes. However, virus that was able to overcome the midgut barriers in HWE females generated stronger infection intensities in their midguts and secondary tissues than in those of LC mosquitoes. 

### 3.2. LACV Vertical Transmission by Ae. albopictus and Ae. aegypti after One or Two Gonotrophic Cycles

To determine the vertical transmission potential of LACV from infected females to their progeny, we assessed the presence of virus in larvae from LC and HWE females that had received an LACV-containing bloodmeal (BM-1), followed by a second non-infectious bloodmeal (BM-2) at 10-days post-BM-1 or just BM-1. In an initial experiment, 30 females of each species oviposited in the same egg cup after each bloodmeal. Eggs were dried and hatched, and the larvae were reared until reaching the L4 stage, when they were sampled in groups of 10. Total RNA was extracted from larva pools and TaqMan qRT-PCR was performed on cDNA to detect the presence of LACV. As somewhat expected [1,41,42], there was no vertical transmission of LACV observed among 15 pools of LC larvae, nor among 27 pools of HWE larvae, which descended from females that had acquired BM-1 only (Table 1). However, the vertical transmission of LACV did occur when females had acquired BM-2, as 4/7 pools (57%) of LC larvae and 1/18 pools (6%) of HWE larvae had detectable virus. Our results show that LACV was vertically transmitted by both mosquito species once they had acquired a second (non-infectious) bloodmeal. The vertical transmission efficiency was significantly lower for HWE than for LC (*p* = 0.0123). 

### 3.3. Ovary Infection Patterns of LACV in HWE and LC Females following Acquisition of a Single Virus-Containing Bloodmeal (BM-1)

**Figure 2:**
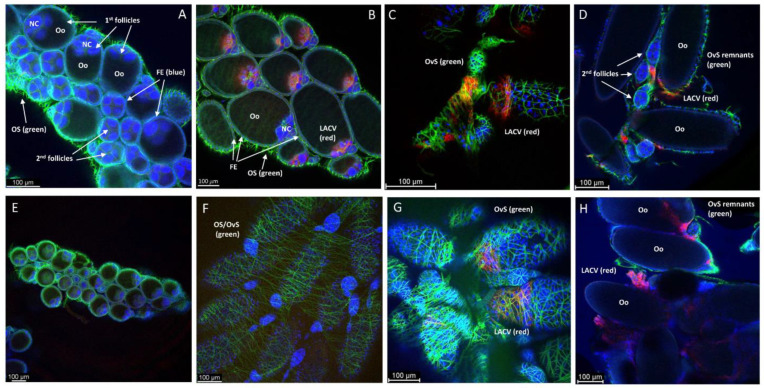
IFCM imaging to detect LACV antigen in *Ae. albopictus* LC and *Ae. aegypti* HWE ovaries after ingestion of a single virus-containing bloodmeal. The virus titer in the bloodmeal was 1.0 × 10^6^ PFUs/mL. (**A**) Ovary of LC at 1 dpi; (**B**) ovary of LC at 2 dpi; (**C**,**D**) developing follicles/oocytes of LC at 4 dpi (before oviposition); (**E**) ovary of HWE at 1 dpi; (**F**) ovary of HWE at 2 dpi; (**G**,**H**) follicles and developing oocytes (before oviposition) of HWE at 4 dpi. Samples were visualized using a Leica TCP SP8 MP confocal microscope and images obtained at 10× and 20× magnification. DAPI (blue): cell nuclei; AlexaFluor 488 (green): actin; AlexaFluor 594 (red): LACV antigen, which was detected using monoclonal antibody 8C2.2 at a 1:200 dilution. Abbreviations: FE: follicular epithelium; NC: nurse cell; Oo: oocyte; OS: ovarian sheath; OvS: ovariole sheath.

**Figure 3:**
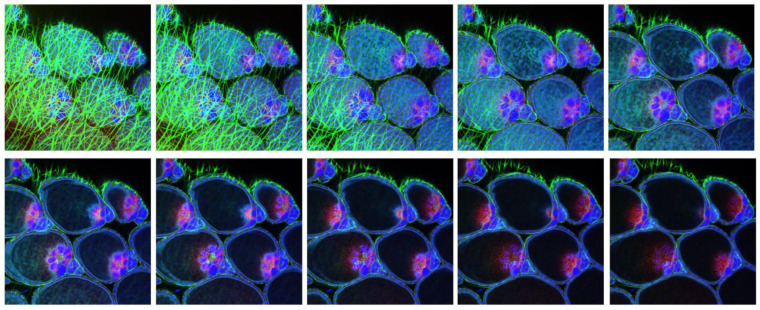
IFCM Z-stack image series showing LACV antigen distribution inside the primary follicles of an *Ae. albopictus* LC female at 2 dpi. Z-stack images were obtained at 20× magnification from the sample preparation shown in Figure 2B using a Leica TCP SP8 MP confocal microscope. The individual slices of the Z-stack were separated from each other by 2.3 mm. DAPI (blue): cell nuclei; AlexaFluor 488 (green): actin; AlexaFluor 594 (red): LACV antigen, which was detected using monoclonal antibody 8C2.2 at a 1:200 dilution.

Following BM-1 ingestion, LC and HWE ovaries were dissected at several time points for the in situ detection of LACV antigen via IFCM. Ten pairs of ovaries were sampled per mosquito species for IFCM staining at 2-, 4-, 7-, and 10-days post-BM-1. As early as 2-days post-BM-1, LACV antigen was detectable in follicular tissue and in the oviduct of a single LC female (Figure 2A,B; Supplemental Figure S1). A Z-stack imaging series of this particular sample suggests that LACV antigen was present inside the primary follicles around the nurse cells (Figure 3). The presence of viral antigen inside most of the primary follicles was further confirmed by 3D image projection (Supplemental Figure S2). Dispersed viral antigen radiated into the zone containing the developing oocyte. A similar observation was not made for other LC or HWE females (Figure 2F), although the sample numbers were rather limited. In three–four individuals of both mosquito species, LACV antigen was visible at 4-days post-BM-1 at the surface of the ovariole sheath enveloping the follicles (Figure 2C,G) and in close proximity to the developing oocytes (Figure 2D,H). Viral antigen was restricted to zones on the ovariole sheath and not encompassing entire ovarioles. In four LC individuals at 7-days and 10-days post-BM-1, and in two HWE individuals at 7-days post-BM-1, viral antigen was found to be closely associated with the ovariole sheath (or other membranous remnants) tightly surrounding the oocytes (Supplemental Figure S3A–D). However, in both mosquito species, no secondary follicles, follicular epithelium, or germarium tissue were observed to contain LACV antigen throughout the time course, which would have been a firm indication for TOT of the virus.

### 3.4. Observing Vertical LACV Transmission by Individual LC and HWE Females

In a further experiment, we sought to assess the LACV ovary infection and vertical transmission rates of individual HWE and LC females, which had acquired two consecutive bloodmeals (Supplemental Table S1; Table 2). We challenged females with (non-infectious) BM-2 at either 7- or 10-days post-BM-1 (containing LACV) to determine whether the incubation time between bloodmeals would impact the vertical transmission rates of the virus. Once individual female mosquitoes of both species had oviposited following the acquisition of BM-2 at 7- or 10-days post-BM-1, the relative proportion of females that oviposited, the number of eggs oviposited by those females, and the larva hatch rates from those eggs were assessed.

Following the acquisition of BM-2 at 7- and 10-days post-BM-1, fewer LC females oviposited (38% and 50%, respectively) than HWE females (67% and 91%, respectively) (Fisher’s Exact Test; *p* = 0.001) (Table 2). Furthermore, LC females laid significantly fewer eggs following the acquisition of BM-2 at 7-days post-BM-1, with a mean number of 11 eggs, compared to 87 eggs oviposited by HWE females (T-test, *p* < 0.0001). These data looked similar when mosquitoes acquired BM-2 at 10-days post-BM-1. Egg hatch rates did not differ significantly between the two mosquito species at both time points of BM-2 acquisition. In accordance with the virus challenge data of Figure 1B, we generally observed relatively low LACV midgut infection rates among HWE females, with 55% and 25% of midguts positive for LACV antigen in mosquitoes that had acquired BM-2 at 7- and 10-days post-BM-1, respectively (Table 2). It needs to be emphasized here that viral infection was determined via the in situ detection of LACV antigen using IFCM (Supplemental Figure S4). By comparison, 92% and 94% of the *Ae. albopictus* LC midguts were positive for LACV antigen under the same regimen. The difference in the overall midgut infection rates (time points 7- and 10-days post-BM-1 combined) between LC and HWE was significant (*p* < 0.0001; Fisher’s Exact Test). We then assessed the ovary infection rate in both species, accounting only for the ovaries from those females that had LACV-infected midguts, as an initial productive midgut infection is a prerequisite (but not a guarantee) for a following ovary infection. LACV infected 33% of the LC ovaries following the acquisition of BM-2 at 7-days post-BM-1, and 40% of the ovaries following the acquisition of BM-2 at 10-days post-BM-1. By contrast, in HWE, the LACV ovary infection rate was significantly higher than in LC, resulting in 83% of virus-infected ovaries in females, which had acquired BM-2 at 7-days post-BM-1, and 67% of virus-infected ovaries in those females that had acquired BM-2 at 10-days post-BM-1 (Fisher’s Exact Test; *p* < 0.05).

One out of the four LC females (25%) that had acquired BM-2 at 7-days post-BM-1 and exhibited an infection of ovarian tissue vertically transmitted LACV to her progeny, as viral genome copies were detected in one larva originating from this female (Table 2). Intriguingly, this female only laid nine eggs from which a single larva hatched (Supplemental Table S1). Similarly, one out of six ovary-infected LC females (17%) from the group that had acquired BM-2 at 10-days post-BM-1 vertically transmitted LACV to her progeny. This female laid 103 eggs, of which 54 hatched (hatch rate: 52%), and in two of those hatched larvae, viral RNA was detected. None of the 10 or 8 HWE mosquitoes that oviposited after receiving BM-2 at 7- and 10-days post-BM-1, respectively, produced any larvae with a detectable LACV infection.

### 3.5. Ovary Infection Patterns of LACV in Individual HWE and LC Females following Acquisition of BM-2 at 7- or 10-Days Post-BM-1

**Figure 4:**
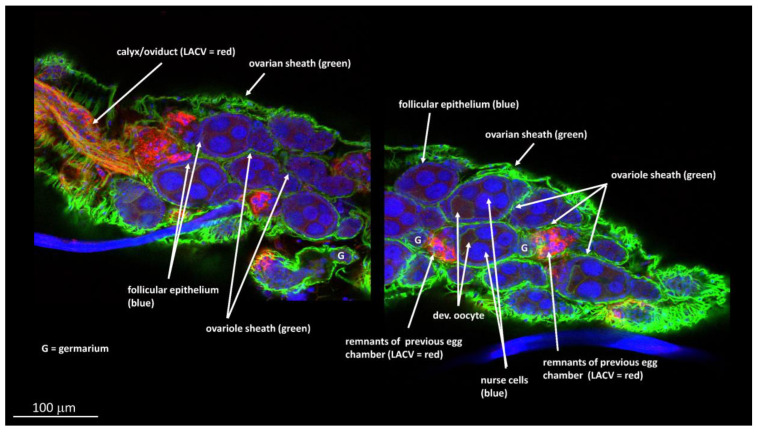
IFCM imaging to detect LACV antigen in an *Ae. aegypti* HWE ovary at 2-days post-ingestion of (non-infectious) BM-2, which was acquired at 7-days post-virus-containing-BM-1. The virus titer in BM-1 was 1.0 × 10^6^ PFUs/mL. The ovary was dissected from HWE female #5 described in Table 2 and Supplemental Table S1. Cell nuclei were stained with DAPI (blue), actin was stained with AlexaFluor 488 (green), and AlexaFluor 594 (red) was used for LACV antigen, which was detected using monoclonal antibody 8C2.2 at a 1:200 dilution. Samples were visualized using a Leica TCP SP8 MP confocal microscope.

**Figure 5:**
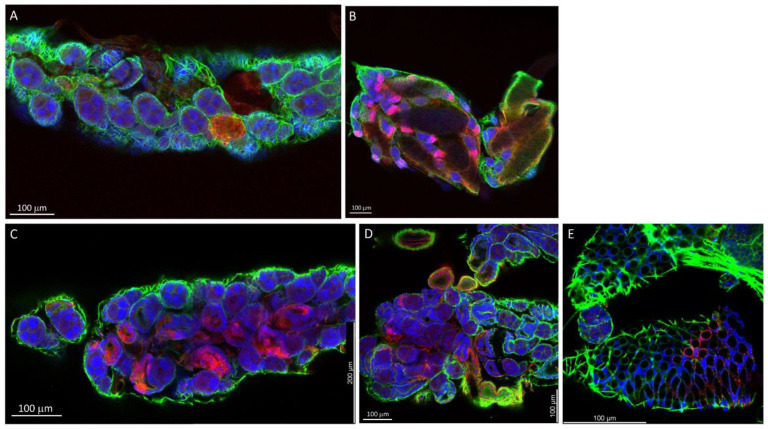
IFCM imaging to detect LACV antigen in *Ae. albopictus* LC and *Ae. aegypti* HWE ovaries at 2-days post-ingestion of (non-infectious) BM-2, which was acquired at 7- or 10-days post-virus-containing-BM-1. The virus titer in BM-1 was 1.0 × 10^6^ PFUs/mL. Ovaries were dissected from those females described in Table 2 and Supplemental Table S1. (**A**) HWE female #4, received BM-2 at 7-days post-BM-1. Follicles of the second gonotrophic cycle are developing. (**B**) HWE female #19, received BM-2 at 10-days post-BM-1. Remaining eggs from the first gonotrophic cycle are visible. Follicles of the second gonotrophic cycle are developing. (**C**) LC female #3, received BM-2 at 7-days post-BM-1. The oocytes from the first gonotrophic cycle have been oviposited. Follicles of the second gonotrophic cycle are developing. (**D**,**E**) LC female #18, received BM-2 at 10-days post-BM-1. In (**E**): viral antigen is detected in ovary-associated tracheal cells. Cell nuclei were stained with DAPI (blue), actin was stained with AlexaFluor 488 (green). AlexaFluor 594 (red) was used for LACV antigen, which was detected using monoclonal antibody 8C2.2 at a 1:200 dilution. Samples were visualized using a Leica TCP SP8 MP confocal microscope.

As shown by IFCM, 7/23 HWE females, which had acquired BM-2 at 7- or 10-days post-BM-1, showed LACV infection of their ovaries (Table 2; Supplemental Table S1). HWE female #5 had widespread LACV antigen staining of her oviduct tissue, through which eggs must pass during oviposition (Figure 4). In this female, LACV antigen was also detected in what appeared to be the remnants of the primary (previous) egg chambers, including parts of the ovariole sheath (as these females had recently oviposited), but not in the ovarian sheath, follicular epithelium, secondary egg chambers, or germarium. From the same group, HWE female #4 showed a strong presence of viral antigen in association with the outside (ovariole sheath) of a single follicle (Figure 5A). HWE female #19 from the group that had acquired BM-2 at 10-days post-BM-1 had strongly detectable viral antigen in the remnants of the primary egg chambers of most of the ovarioles (Figure 5B). We observed similar LACV infection patterns in the ovaries of 10/29 LC females that had acquired BM-2 at 7- or 10-days post-BM-1 (Table 2; Supplemental Table S1). As exemplified for females #3 and #18, which received BM-2 at 7- or 10-days post-BM-1, respectively, bright LACV antigen staining was found associated with the ovariole sheath and within the remnants of the primary egg chambers (Figure 5C,D). In LC female #18, we also observed strong LACV antigen accumulation around (and within) the ovarian tracheal cells (Figure 5E). Antigen staining was not observed in the ovarian sheath, the follicular epithelium, or germarium tissue. Two of these ten females produced three progeny larvae that were LACV-infected (Table 2).

**Figure 6:**
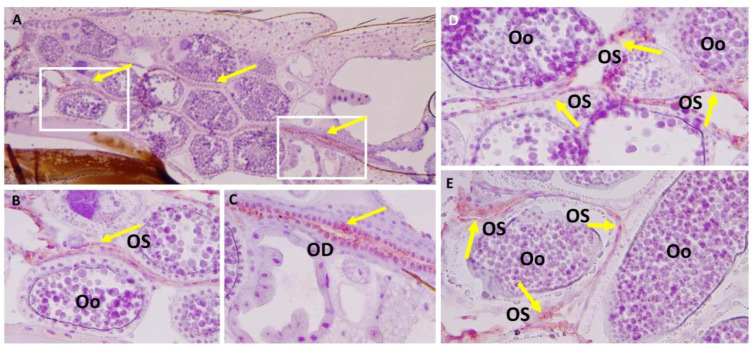
Immunohistochemistry (IHC) assay to detect LACV infection in reproductive tissues of *Ae. aegypti* HWE mosquitoes, which had ingested two consecutive bloodmeals. Females ingested (non-infectious) BM-2 at 10-days post-LACV-containing-BM-1 (titer in the bloodmeal: 1.3 × 10^6^ PFUs/mL). Five HWE mosquitoes were collected for sectioning and IHC staining at 24 h post-BM-2. Viral antigen was detected in two of the five females. (**A**) LACV antigen staining (dark red, yellow arrows) is visible throughout the ovaries of a female at 24 h post-BM-2. White squares represent areas magnified in B and C images. (**B**) LACV antigen staining is restricted to the ovariole sheath (OS) between the oocytes (Oo). (**C**) The oviduct (OD) was found to be strongly LACV-infected. (**D**,**E**) In another *Ae. aegypti* HWE mosquito sampled at 48 h post-BM-2, LACV antigen was detected at the ovariole sheath but not within the developing oocytes. LACV antigen was detected using monoclonal antibody 8C2.2 at a 1:200 dilution.

**Figure 7:**
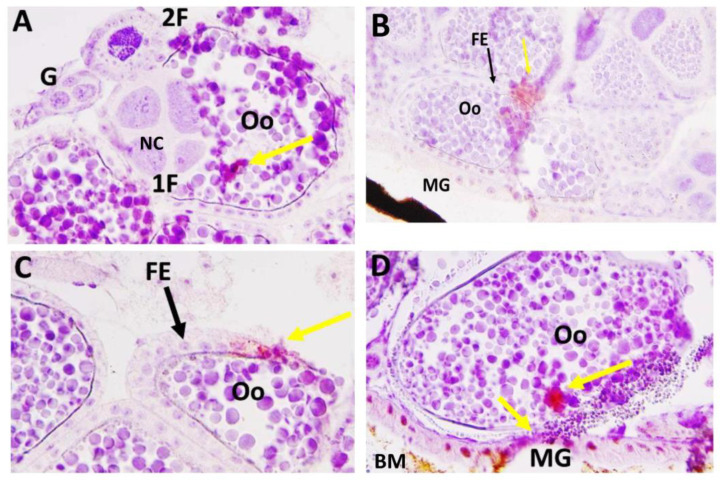
Immunohistochemistry (IHC) assay to detect LACV infection in reproductive tissues of *Ae. albopictus* LC mosquitoes, which had ingested two consecutive bloodmeals. Females ingested (non-infectious) BM-2 at 10-days post-LACV-containing-BM-1 (titer in the bloodmeal: 1.3 × 10^6^ PFUs/mL). Six LC females were collected for sectioning and IHC staining at 24 h post-BM-2. Viral antigen was detected in four of the six females. (**A**–**D**) Distinct traces of LACV antigen (yellow arrows) are visible inside oocytes (Oo). FE: Follicular epithelium; G: germarium; 1F: primary follicle; 2F: secondary follicle; NC: nurse cell. In (**D**): viral antigen is associated with a small section of the midgut epithelium (MG) adjacent to the oocyte. The partly digested bloodmeal (BM) is visible. LACV antigen was detected using monoclonal antibody 8C2.2 at a 1:200 dilution.

We then performed IHC assays using the LACV monoclonal antibody on paraffin-embedded whole-body sections of HWE and LC mosquitoes to gain another view of the viral infection patterns inside the ovaries. These mosquitoes were not any of those listed in Supplemental Table S1 or Table 2. In two out of five HWE females, which had received BM-2 at 10-days post-BM-1, we observed a strong presence of LACV antigen at 24 h post-BM-2. As shown for the two HWE mosquitoes, viral antigen was strongly associated with the ovariole sheath (Figure 6A,B,D,E) and oviduct (Figure 6A,C). We did not observe viral antigen within any follicles, including the developing oocytes. Under a similar infection regimen, we observed the presence of LACV antigen in four out of six LC females. As shown for the four females, distinct traces of LACV antigen were detected in individual (developing) oocytes (Figure 7A–D). One of the LACV-positive ovaries was adjacent to the midgut epithelium, which also contained viral antigen (Figure 7D). The detection of LACV antigen inside oocytes is suggestive of a TOT mode.

## 4. Discussion

The objective of this study was to compare the vertical LACV transmission potential between *Ae. albopictus* and *Ae. aegypti*, and to visualize the ovarian infection patterns in both mosquito species. In a separate experiment, systemic mosquito infection was analyzed by quantifying the LACV infection levels in individual tissues of both mosquito species via plaque assays. Our results show that LACV prevalence was significantly higher in the midguts, head tissue, and ovaries of *Ae. albopictus* in comparison to *Ae. aegypti*, which exhibited relatively strong midgut infection and midgut escape barriers against the virus. *Ae. albopictus*, in contrast to *Ae. aegypti*, has been described as a natural vector for LACV [11,12]. Our results suggest that the presence/absence of midgut infection and escape barriers are important factors determining the vector competence for the virus in both mosquito species. Interestingly, the virus replication/intensity of infection was significantly higher in the midguts and secondary tissues of those *Ae. aegypti* mosquitoes that became infected when compared to *Ae. albopictus*. In an earlier study, Sim et al. (2013) conducted an extensive genome-wide gene expression analysis among various *Ae. aegypti* strains correlating the immunity-related gene expression levels in those mosquitoes to variable intensities of infection with dengue 2 virus [43]. Accordingly, we can speculate that LACV triggered antiviral immune responses, such as RNAi, Toll, Imd, and/or the JAK-STAT pathway, more strongly in *Ae. albopictus* LC than in *Ae. aegypti* HWE. These observations are in agreement with earlier studies showing that *Ae. albopictus* was more competent for the virus than *Ae. aegypti* [44,45]. Moreover, we showed that LACV had a similar replication dynamic in both mosquito species, as has been reported for CHIKV and MAYV in *Ae. aegypti*, in which these two alphaviruses had an extrinsic incubation period as short as two days [46,47,48].

Similar to the horizontal mode of virus transmission, vertical virus transmission efficiencies can vary widely among particular arbovirus–mosquito combinations [29,44,49,50,51]. In our study, the vertical transmission potential of LACV was at least three-fold lower for *Ae. albopictus* LC compared to earlier observations made by Hughes et al. (2006) [44]. For *Ae. aegypti* HWE, we observed the vertical transmission of LACV only in 1/18 larva pools, each consisting of 10 larvae. Vertical arbovirus transmission by mosquitoes can be affected by several factors, such as the viral and mosquito taxa, number of gonotrophic cycles, incubation period between gonotrophic cycles, and environmental factors, such as the temperature/season [49,50,51,52,53,54]. Hughes et al. (2006) [44] used similar strains of *Ae. albopictus* (LC) and *Ae. aegypti* (RexD, the parental strain of the HWE eye-pigment-deficient mutant) but used a different LACV strain (Human 78) and provided two consecutive non-infectious bloodmeals instead of just one before collecting any vertical transmission data. They also used a different viral detection method based on head squashes followed by immunostaining. It is not obvious which of these factors might have contributed most strongly to the divergent results of our study regarding the vertical transmission of LACV by *Ae. aegypti* and *Ae. albopictus*. Furthermore, in our work, the incubation time period between BM-1 and BM-2 (7 or 10 days) did not have a significant impact on the vertical transmission outcome [52].

In accordance with earlier observations by others, we did not see any vertical transmission of the virus to the F1 progeny in those females that had acquired just a single LACV-containing bloodmeal [1,41]. The authors of these studies observed that LACV was not detected in ovarian tissue before 7 dpi via artificial virus-containing bloodmeals and claimed that, by that timepoint, first-cycle eggs had been oviposited already or were in their late stage of development, which would render them refractory to infection. In our study, LACV was detected in the follicular tissue of an *Ae. albopictus* female as early as 2 dpi via a single bloodmeal. Systemic viral infection of the (primary) follicles is a strong indication for TOT [38,55,56], and we observed this during the first gonotrophic cycle. However, at this point, we do not know whether this single observation represented a rare event or whether it would occur more frequently. In the future, a larger number of LACV-challenged *Ae. albopictus* females need to be analyzed for follicular tissue infection. It can be speculated that females showing follicular tissue infection following the ingestion of a single infectious bloodmeal would generate virus-infected progeny unless the virus is damaging or killing the developing oocytes. At later time points post-infection with LACV (based on a single gonotrophic cycle), we did not observe any follicular tissue infection. Instead, at 4 dpi, viral antigen was associated in both mosquito species with the ovariole sheath surrounding the follicles and (at 7 days dpi) with its remnants that were (still) attached to the maturing oocytes. There may be just a very narrow time window early during follicular development when LACV is able to infect the follicular tissue.

When *Ae. triseriatus* was infected with the same LACV strain as used in our study via a single infectious bloodmeal, viral antigen was detected in the follicular epithelium, nurse cells, and oocytes of most of the females tested, indicative of TOT [38]. Similarly, Bergren et al. (2021) [57] detected viral antigen in those same tissues of *Culex tarsalis* females, which had been orally challenged with a single bloodmeal containing RVFV. Furthermore, the intrathoracic injection of *Ae. mcintoshi* with RVFV followed by the provision of two consecutive non-infectious bloodmeals caused the infection of the same reproductive tissues in females [55]. Based on their in situ detection studies with SAV in *Ae. albopictus*, Tesh and Cornet (1981) [56] speculated that the virus first enters the ovaries via the oviduct and ovariole sheath before subsequently infecting the follicular epithelium, oocytes, and nurse cells of primary follicles. Similarly, it was suggested that RVFV within the hemocoel of infected *Ae. mcintoshi* females would infect the ovarian and ovariole sheaths followed by virus entry into the follicular epithelium [55]. The authors also emphasized the role of RVFV-infected ovarian tracheal cells acting as a possible conduit for the virus on its route from the hemocoel to the ovaries. The involvement of tracheal cells in the ovary infection process has been also hypothesized for LACV in *Ae. triseriatus* [38]. Indeed, in our study, we observed tracheal cells that were associated with the ovarian/ovariole sheath in *Ae. albopictus* LC to be LACV-infected, supporting the idea that the tracheal cell route may be relevant for ovary infection with bunyaviruses.

In all those experimental mosquito–arbovirus combinations that resulted in TOT of the virus and in which ovarian infection patterns were assessed in situ, the follicular epithelium and/or developing oocytes of the mosquitoes were observed to be virus-infected [31,38,55,56,57]. We did not find the follicular epithelium infected with LACV in any of our samples, although we found, at least in one sample, follicular nurse cells associated with viral antigen. This suggests that the virus does not enter the follicular tissue by traversing the infected ovariole sheath; otherwise, the follicular epithelium would have been expected to contain viral antigen. Instead, LACV seems to enter the follicular tissue early during development via the oviduct/calyx. This route of infection seems to be sufficient to infect a few oocytes, as observed by IHC. Regardless, the exact route that LACV uses to enter the follicular tissue needs to be further investigated and repeatedly observed before firm conclusions can be drawn.

When both mosquito species had ingested two consecutive bloodmeals, the first one of which contained LACV, viral antigen was found abundantly present in remnants of the primary egg chambers that likely also contained fragments of the ovariole sheath. Viral antigen was not clearly detectable inside primary or secondary follicles, while the oviducts of both mosquito species were virus-infected. Detection of LACV in individual eggs from several LC females suggests that TOT does occur in *Ae. albopictus*. The distinct small foci of LACV infection inside the oocytes of LC females resembled those distinct traces of RVFV antigen detected in the oocytes of *Ae. mcintoshi*, potentially causing mild or latent infection in those, which could then result in infected progeny larvae hatching from these eggs [55]. Furthermore, single progeny larvae from such infected *Ae. albopictus* females were found to be LACV-infected. Thus, in at least some *Ae. albopictus* LC females, LACV might be vertically transmitted via TOT, although with relatively low efficiency. In *Ae. aegypti* HWE, however, we did not find follicular tissue or oocytes to be infected with the virus. Instead, ovarian sheaths and oviducts contained viral antigen. Similar observations had been made earlier for the flaviviruses Japanese encephalitis virus, St. Louis encephalitis virus, and DENV, which were abundantly detected in the ovarian sheaths and oviducts of experimentally infected *Ae. albopictus* females, but not so in their follicular tissues [26]. In his 1987 paper, Rosen [25] describes this type of ovarian infection pattern as an indication of the transovum transmission of virus rather than TOT. Transovum transmission is considered widespread among mosquito-borne flaviviruses and alphaviruses. It represents a much less efficient mode of vertical virus transmission in which the developed oocyte on its surface picks up virus from the infected oviduct (or ovariole sheath) during the oviposition process. Occasionally, this then causes the infection of the progeny larva developing inside the egg, as was found in our study. Variations in transovum transmission efficiencies, as observed among different mosquito–arbovirus combinations, could be due to varying levels of infection of the females’ genital tracts in each situation [58].

However, LACV, so far, has been primarily associated with TOT in *Ae. triseriatus* [1] and, in one study, also with *Ae. albopictus* and *Ae. aegypti* [44]. Typically, TOT in mosquitoes leads to much higher progeny infection rates (as much as >80% have been reported), which eventually can develop into stabilized infections [59]. Tesh and Shroyer (1980) [50] and Shroyer (1986) [51] discussed and investigated the possibility of genetic factors from the individual female mosquito controlling TOT, which perhaps are similar to those controlling the vertical transmission of Sigma virus (*Rhabdoviridae*) in *Drosophila* [1]. These genetic factors are not evenly expressed among all individuals of a population. However, chronically SAV-infected lines of *Ae. albopictus* were developed based on the recurrent selection of those females that highly efficiently transmitted the virus via TOT, resulting in filial infection rates of ~100%. QTL mapping in two LACV-infected *Ae. triseriatus* populations, one of them strongly supporting TOT and the other one weakly supporting TOT, suggested the presence of a genetic factor in the form of at least one dominant heterozygous allele promoting TOT, whereas a recessive homozygous allele would account for the TOT refractory phenotype [60]. The authors then speculated that a transmembrane protein (i.e., a viral receptor at the follicular tissue) could account for such a TOT factor. Following this scenario, it then appears that, in our study, none of the *Ae. aegypti* HWE females and only a few of the *Ae. albopictus* LC females possessed the genetic factor(s) supporting the TOT of the 1960 strain of LACV.

## 5. Conclusions

*Ae. aegypti* and *Ae. albopictus* significantly differed in their vector competences for LACV. In *Ae. aegypti*, the virus was confronted with strong midgut infection and midgut escape barriers, which were absent in *Ae. albopictus*. Productive LACV infection of the midgut was a prerequisite (but not a guarantee) for the following infection of ovarian tissue. Following the ingestion of a single LACV-containing bloodmeal, two different ovarian infection patterns were observed: (i) infection of the ovariole sheath outside the follicles; (ii) viral infection inside the primary follicles around the nurse cells (in a single *Ae. albopictus* female). Following the ingestion of two consecutive bloodmeals, LACV antigen was detected in a few oocytes from *Ae. albopictus*, while vertical LACV transmission was observed for both mosquito species. However, the observed vertical transmission rates were much lower than previously reported for (other) TOT systems. It is likely that vertical LACV transmission occurred via the transovum route instead of the TOT route in those females in which the ovariole sheath, but not the follicular tissue or oocytes, were LACV-infected. Neither germarium, secondary follicles, or follicular epithelium were observed to be LACV-infected in either mosquito species. These tissues have been earlier described as playing a key role in arbovirus TOT.

## Figures and Tables

**Table 1:** Vertical transmission among pooled F1 larvae from *Aedes* mosquitoes, which received an LACV-containing bloodmeal (BM-1) only or a second non-infectious bloodmeal (BM-2) at 10-days post-BM-1.

Mosquito	Number of Bloodmeals ^1^	LACV-Infected Larva Pools (%) ^2^
*Ae. albopictus* LC	BM-1 only	0/15 (0)
	BM-2: 10-days post-BM-1	4/7 (57%)
*Ae. aegypti* HWE	BM-1 only	0/27 (0)
	BM-2: 10-days post-BM-1	1/18 (6%)

^1^ Mated one-week-old females *(n* = 30) received an initial bloodmeal (BM-1) containing 10^6^ PFUs/mL LACV and their progeny were assayed at 4-days post-BM-1 or the females received a second non-infectious bloodmeal at 10-days post-BM-1 and their progeny were assayed at 4-days post-BM-2. ^2^ A total of 10 larvae were combined per pool and assayed via TaqMan qRT-PCR for the presence of LACV.

**Table 2:** Tissue-specific infection and vertical transmission profiles of LACV by individual *Ae. aegypti* and *Ae. albopictus* females following ingestion of a second bloodmeal (BM-2) at 7- or 10-days post-initial-infectious-bloodmeal (BM-1).

Mosquito	Days between BM-1 and BM-2 Ingestion ^1^	Midgut Infection Rate (%) ^2^	Ovary Infection Rate (%) ^3^	Proportion of Females that Oviposited (%)	Average No. of Eggs Oviposited per Female ^4^	Hatch Rate in %	VTR ^5^
*Aedes albopictus* LC	7	12/13 (92%)	4/12 (33%)	5/13 (38%)	11 ± 2.2	32 ± 15.4	1/4 (25%)
	10	15/16 (94%)	6/15 (40%)	8/16 (50%)	18 ± 8.9	48 ± 12.8	1/6 (17%)
*Aedes aegypti* HWE	7	6/11 (55%)	5/6 (83%)	10/11 (91%)	87 ± 9.9	64 ± 9.3	0/5 (0)
	10	3/12 (25%)	2/3 (67%)	8/12 (67%)	95 ± 12.7	45 ± 11.4	0/2 (0)

^1^ Initial bloodmeal contained 10^6^ PFUs/mL LACV. ^2^ Midgut infection rate: number of midguts infected divided by the number of midguts assayed; *p* < 0.0001. The presence of viral antigen in midguts was assayed via IFCM. ^3^ Ovary infection rate: number of ovaries infected divided by the number of midguts infected (females that did not have a midgut infection also had no ovary infection); *p* = 0.0551. The presence of viral antigen in ovaries was assayed via IFCM. ^4^ Oviposition was monitored for 5 days; *p* = 0.0227. ^5^ VTR (vertical transmission rate): number of females transmitting LACV to at least 1 progeny divided by the number of ovary-infected females. Individual larvae were assayed for the presence of LACV via TaqMan qRT-PCR.

## Data Availability

The data presented in this study are available on request from the corresponding author.

## Supplementary Materials

The following supporting information can be downloaded at https://www.mdpi.com/article/10.3390/insects14070601/s1, Figure S1: IFCM image showing both ovaries and the oviduct of the *Ae. albopictus* LC-2 dpi sample shown in Figure 2B and Figure 3; Figure S2: Converted 3D-projections derived from the ovary sample shown in Figure 2B, Figure 3, and Supplemental Figure S1 (*Ae. albopictus* LC at 2 dpi); Figure S3: IFCM imaging to detect LACV antigen in *Ae. albopictus* LC and *Ae. aegypti* HWE ovaries after ingestion of a single virus containing bloodmeal; Figure S4: IFCM images showing the presence of LACV antigen in midguts of (A) *Ae. albopictus* LC and (B) *Ae. aegypti* HWE at 4 days post-LACV containing bloodmeal; Table S1: LACV infection status, fecundity, and vertical transmission of individual *Ae. albopictus* LC and *Ae. aegypti* HWE females after ingestion of a LACV containing BM-1 followed by a non-infectious BM-2 at 7 or 10 days post-BM-1.
