# Supplementary material for: Detection of La Crosse Virus In Situ and in Individual Progeny to Assess the Vertical Transmission Potential in Aedes albopictus and Aedes aegypti
Source: Insects. 2023 Jul 3;14(7):601. doi: 10.3390/insects14070601 (PMC10380845; doi:10.3390/insects14070601)
Supplement: Supplementary file 1 [file insects-14-00601-s001.zip › Supplemental Figures S1-S4.pdf]

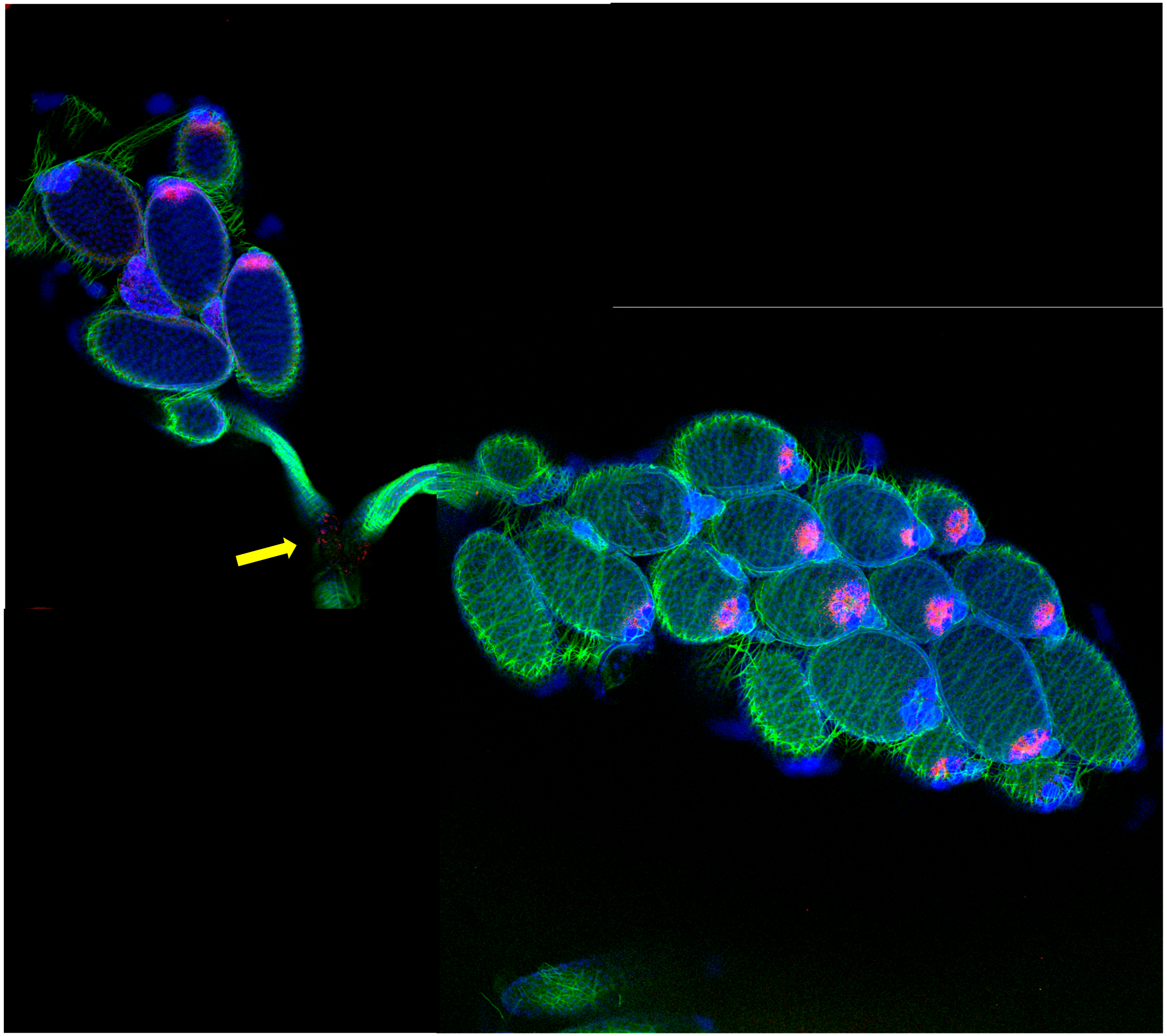

Supplemental Figure S1: IFCM image showing both ovaries and the oviduct of the *Ae. albopictus* LC-2 dpi sample shown in Figure 2B and Figure 3. DAPI (blue) = cell nuclei, AlexaFluor 488 (green) = actin. AlexaFluor 594 (red) = LACV antigen, which was detected using monoclonal antibody 8C2.2 at a 1:200 dilution. The yellow arrow points towards LACV antigen in the oviduct. Samples were visualized using a Leica TCP SP8 MP confocal microscope.

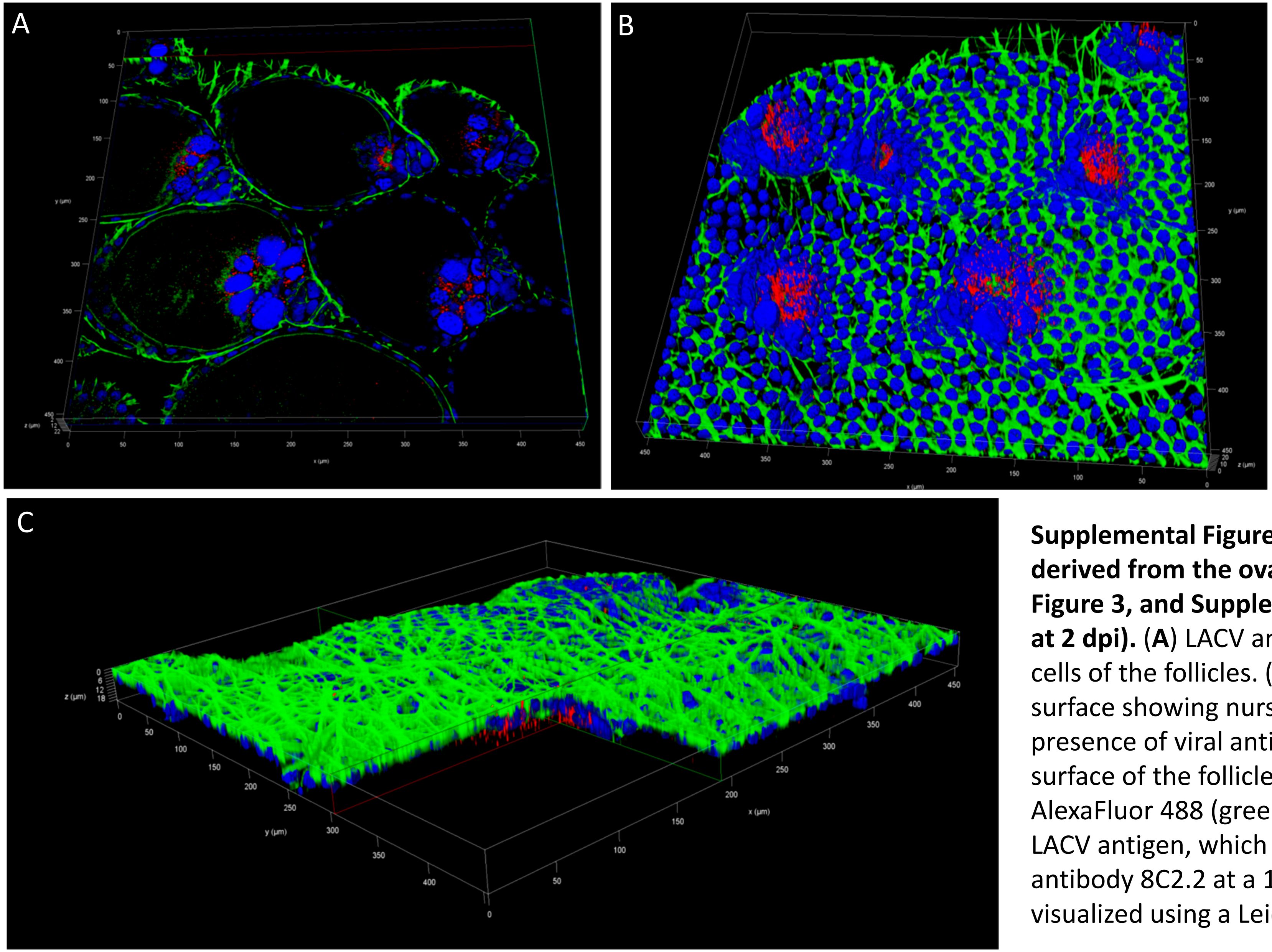

Supplemental Figure S2: **Converted 3D-projections derived from the ovary sample shown in Figure 2B, Figure 3, and Supplemental Figure 1 (*Ae. albopictus* LC at 2 dpi).** (**A**) LACV antigen is associated with the nurse cells of the follicles. (**B**) View towards the inner follicular surface showing nurse cells, ovariole sheath, and the presence of viral antigen. (**C**) View towards the outer surface of the follicles. DAPI (blue) = cell nuclei, AlexaFluor 488 (green) = actin. AlexaFluor 594 (red) = LACV antigen, which was detected using monoclonal antibody 8C2.2 at a 1:200 dilution. Samples were visualized using a Leica TCP SP8 MP confocal microscope.

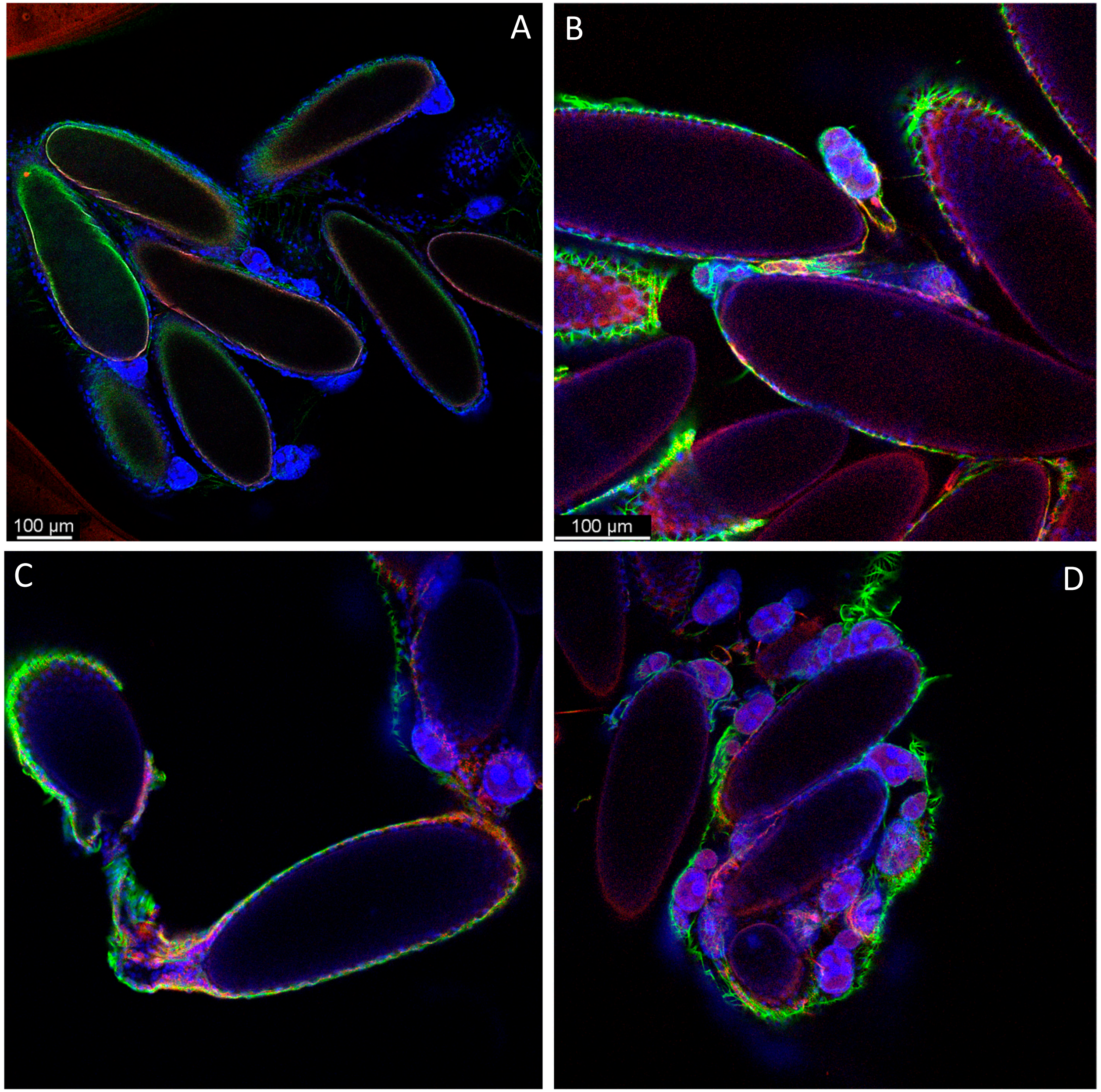

Supplemental Figure S3: IFCM imaging to detect LACV antigen in *Ae. albopictus* LC and *Ae. aegypti* HWE ovaries after ingestion of a single virus containing bloodmeal. The virus titre in the bloodmeal was 1x10^6 PFU/ml. (**A**) oocytes of HWE at 7 dpi; (**B**) oocytes of LC at 7 dpi; (**C, D**) oocytes of LC at 10 dpi before oviposition. Samples were visualized using a Leica TCP SP8 MP confocal microscope and images obtained at 10x magnification. DAPI (blue) = cell nuclei, AlexaFluor 488 (green) = actin. AlexaFluor 594 (red) = LACV antigen which was detected using monoclonal antibody 8C2.2 at a 1:200 dilution.

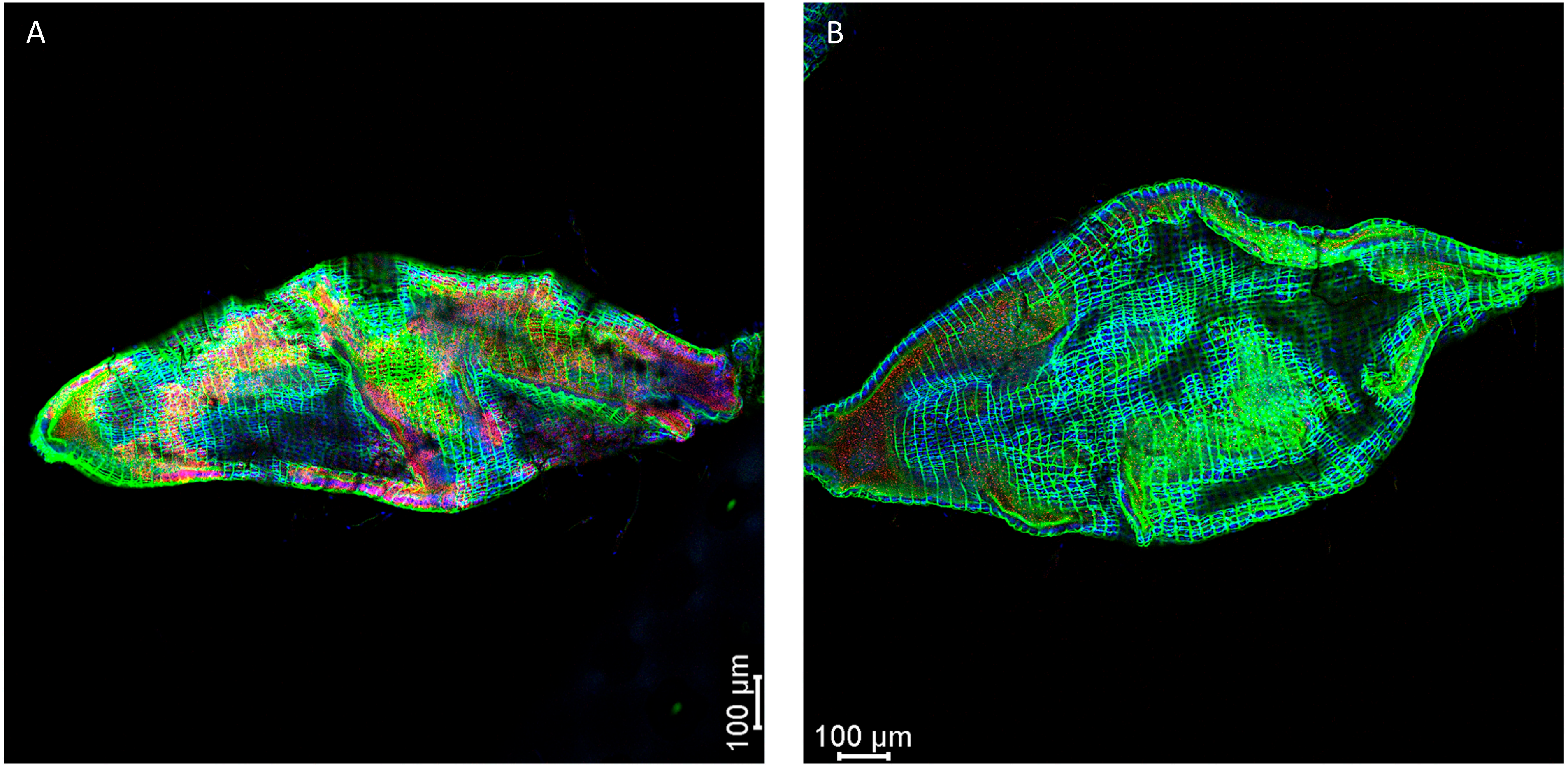

Supplemental Figure S4: IFCM images showing the presence of LACV antigen in midguts of (A) *Ae. albopictus* LC and (B) *Ae. aegypti* HWE at 4 days post-infectious bloodmeal. DAPI (blue) = cell nuclei, AlexaFluor 488 (green) = actin. AlexaFluor 594 (red) = LACV antigen, which was detected using monoclonal antibody 8C2.2 at a 1:200 dilution. Samples were visualized using a Leica TCP SP8 MP confocal microscope.
